# Development and validation of a postoperative risk model for esophageal squamous cell carcinoma after neoadjuvant immunochemotherapy

**DOI:** 10.3389/fmed.2025.1608313

**Published:** 2025-08-04

**Authors:** Hai Zhang, Cui Li, Jiangbo Lin, Xihao Xie, Fengyuan Peng, Caihou Feng, Weibi Che, Jiawei Huang, Bomeng Wu

**Affiliations:** ^1^Department of Thoracic Surgery, Gaozhou People’s Hospital, Gaozhou, China; ^2^Department of Thoracic Surgery, Fujian Medical University Union Hospital, Fuzhou, China

**Keywords:** esophageal squamous cell carcinoma, neoadjuvant immunochemotherapy, recurrence risk model, minimally invasive esophagectomy, pathological N stage, nerve invasion, survival outcomes

## Abstract

**Background:**

Esophageal squamous cell carcinoma (ESCC) remains a highly aggressive malignancy with a significant risk of recurrence, even after curative treatment. While neoadjuvant immunochemotherapy (nICT) combined with minimally invasive esophagectomy (MIE) has shown promise in improving outcomes for patients with locally advanced, resectable ESCC, the factors contributing to early postoperative recurrence remain unclear. This study aims to identify high-risk factors for short-term recurrence and develop a predictive model for recurrence in patients with locally advanced, resectable ESCC treated with nICT followed by MIE (McKeown approach).

**Methods:**

Patients with locally advanced, resectable ESCC who underwent nICT followed by MIE at Gaozhou People’s Hospital between 1 January 2019, and 1 January 2022, were consecutively included in the training set. Patients who received the same treatment at Union Hospital of Fujian Medical University during the same period were included as the validation set. A recurrence prediction model was developed based on these cohorts.

**Results:**

A total of 362 patients treated with nICT were included, including 218 in the training set and 144 in the validation set. Least absolute shrinkage and selection operator regression identified the 10 most significant variables associated with recurrence: smoking history, drinking history, diarrhea, number of lymph nodes dissected, number of lymph node dissection stations, pathological N (pN) stage, pathological TNM stage, tumor regression grade, nerve invasion, and postoperative arrhythmia. Multivariate regression analysis further identified pN+ and nerve invasion as independent high-risk factors for recurrence. The recurrence prediction model demonstrated strong discriminatory ability, with an area under the curve of 0.92 in the training set and 0.91 in the validation set at 3 years postoperatively. Survival analysis showed a statistically significant difference (*p* < 0.05) in the 3-year overall survival and recurrence-free survival between risk groups. In the low-risk group, postoperative adjuvant therapy did not provide a survival benefit; in the high-risk group, it significantly improved outcomes.

**Conclusion:**

Patients with locally advanced ESCC treated with nICT followed by MIE who have a high pN stage and pathological evidence of nerve invasion may benefit from intensified adjuvant therapy to improve long-term survival.

## 1 Introduction

For patients diagnosed with locally advanced, surgically resectable esophageal squamous cell carcinoma (ESCC), neoadjuvant therapy combined with surgery is the standard treatment. However, even after radical esophageal cancer surgery, the risk of recurrence remains high at 30%–50%, significantly affecting patient survival outcomes (1). Recurrence patterns are classified based on postoperative recurrence—local, regional, or distant metastasis (2)—and by timing, as either early or late recurrence (3, 4). Although standard neoadjuvant chemoradiotherapy (nCRT) provides good local control, contemporary clinical data indicate that the incidence of postoperative recurrence persists at concerningly elevated levels, approximating 40% (5). Notwithstanding the attainment of pathological complete response (PCR), 20%–30% still experience recurrence (6). Zhou et al. (7) analyzed 282 patients with ESCC who underwent nCRT followed by surgery and developed three prediction models incorporating pathological factors. Their findings indicated that a clinical prediction model integrating lymph node tumor regression grade (TRG) factors demonstrated high predictive accuracy in identifying patients with elevated recurrence risk following neoadjuvant therapy (7). Similarly, Qiu et al. (6) included 206 patients with ESCC treated with nCRT who achieved PCR and constructed a novel prediction model integrating imaging and clinical data. This model improved the accuracy of recurrence risk assessment in postoperative patients with ESCC. These findings underscore the necessity of developing precise recurrence prediction models to enhance patient prognosis and guide individualized treatment strategies (7).

Immune checkpoint inhibitors have shown promising efficacy in advanced esophageal cancer, and several clinical trials have confirmed that neoadjuvant immunochemotherapy (nICT) improves PCR rates and prolongs progression-free survival in locally advanced, resectable ESCC. However, due to patient heterogeneity, some individuals remain at risk of early recurrence (8–11). Currently, prediction tools for assessing recurrence risk after nICT remain inadequate. The present investigation endeavored to construct and externally validate a prognostic model for evaluating recurrence risk following surgical intervention in ESCC patients receiving nICT. The findings will provide an evidence-based foundation for optimizing postoperative adjuvant treatment strategies involving nICT and surgery, establishing a theoretical framework for targeted intervention and clinical trial design, as well as advancing the individualized development of nICT-based treatment modalities.

## 2 Materials and methods

### 2.1 Study participants

This retrospective cohort study included patients with ESCC who received nICT at Gaozhou City People’s Hospital and Fujian Medical University Union Hospital between 1 January 2019, and 1 January 2022.

Inclusion criteria: ➀ age 18–75 years, both male and female, with an ECOG score of 0–1; ➁ pathologically confirmed ESCC; ➂ preoperative confirmation of resectable, locally advanced ESCC and treatment with nICT; and ➃ availability of complete clinical data. Exclusion criteria: ➀ presence of other pathological types of esophageal malignancies; ➁ severe comorbidities; and ➂ concurrent diagnosis of other malignant tumors.

### 2.2 Ethics approval

This study was conducted in accordance with the principles of the Declaration of Helsinki. Ethical approval and a waiver of informed consent were obtained from the Ethics Committee of Gaozhou City People’s Hospital and the Institutional Review Board of Union Hospital, Fujian Medical University.

### 2.3 Observation indicators

The demographic characteristics of the enrolled patients included region (urban or rural), gender, and age. Anthropometric data such as body mass index and weight loss were recorded. Concomitant comorbidities (hypertension, diabetes mellitus, and coronary artery disease) were documented. Personal history variables, including smoking and alcohol consumption, were assessed. Tumor characteristics were documented based on location, classified as the upper, middle, or lower esophagus. The incidence of adverse events during neoadjuvant therapy was evaluated, including anemia, leukopenia, neutropenia, thrombocytopenia, elevated aspartate aminotransferase, elevated alanine aminotransferase, elevated bilirubin, elevated creatinine, vomiting, diarrhea, immune-related pneumonia, immune-related dermatitis, and esophageal perforation. Treatment-related factors, such as the chemoimmunotherapy course and the clinical TNM stage, were recorded. Perioperative data included the surgical approach, operative time, and intraoperative blood loss. Lymph node assessment involved documenting the number of lymph nodes dissected and the number of lymph node clearance stations. Surgical outcomes were classified based on the type of resection (R0 or R1), post-neoadjuvant therapy pathological TNM (pTNM) stage, TRG, presence of nerve invasion, and vascular invasion. Postoperative recovery parameters included duration of chest tube drainage, length of hospital stay, and incidence of postoperative complications such as arrhythmia, anastomotic fistula, and pneumonia. Follow-up data were also collected to assess patient outcomes.

### 2.4 Statistical methods

Between-group differences in categorical variables were analyzed using the χ^2^ test or Fisher’s exact test. Normally distributed continuous variables were compared using the independent *t*-test, whereas non-normally distributed continuous variables were analyzed using the Mann–Whitney *U* test. Covariate selection was executed via least absolute shrinkage and selection operator (LASSO) regression implemented in the glmnet package (v4.1-8), with hyperparameter λ optimized through 10-fold cross-validation minimizing mean squared error (MSE). Statistically significant predictors subsequently underwent multivariate backward stepwise logistic regression modeling to ascertain independent determinants. Model predictive accuracy was evaluated through receiver operating characteristic (ROC) curve analysis and corresponding area under the curve (AUC) quantification. Analytical procedures were implemented in R (v4.4.3).

## 3 Results

### 3.1 Baseline characteristics of study participants

This retrospective study included 218 patients from Union Hospital of Fujian Medical University, forming the training set, and 144 patients from Gaozhou People’s Hospital, forming the validation set. Baseline data for patients in both groups included age, gender (1 = male, 2 = female), region (1 = urban, 2 = rural), weight loss in last 3 months, smoking index (0 = non-smoking, 1 = smoking index of 1–400 cigarettes per year, 2 = smoking index of 400–800 cigarettes per year, 3 = smoking index of more than 800 cigarettes per year), tumor location (1 = upper segment, 2 = middle segment, 3 = lower segment), and clinical TNM stage (2 = stage II, 3 = stage III, 4 = stage IV). Additionally, alcohol consumption, hypertension, diabetes mellitus, and coronary artery disease were recorded (0 = no, 1 = yes). No statistically significant intergroup disparities were observed in baseline demographic or clinical characteristics (Table 1).

**TABLE 1:** Comparison of baseline data between the training set and the validation set.

Characteristic	Group		*p*-Value
	Train set, *N* = 218	Validation set, *N* = 144	
**Age**	60.5 ± 6.6	59.9 ± 6.1	0.330
**Sex**			0.994
1	171 (78.4%)	113 (78.5%)	
2	47 (21.6%)	31 (21.5%)	
**Registration**			0.719
1	173 (79.4%)	112 (77.8%)	
2	45 (20.6%)	32 (22.2%)	
**Weight loss**			0.920
0	196 (89.9%)	129 (89.6%)	
1	22 (10.1%)	15 (10.4%)	
**BMI**	22.29 ± 12.71	21.52 ± 2.78	0.393
**Smoking**			0.806
0	99 (45.4%)	67 (46.5%)	
1	19 (8.7%)	16 (11.1%)	
2	39 (17.9%)	26 (18.1%)	
3	61 (28.0%)	35 (24.3%)	
**Drinking**			0.930
0	155 (71.1%)	103 (71.5%)	
1	63 (28.9%)	41 (28.5%)	
**Hypertension**			0.499
0	182 (83.5%)	124 (86.1%)	
1	36 (16.5%)	20 (13.9%)	
**Diabetes**			0.813
0	203 (93.1%)	135 (93.8%)	
1	15 (6.9%)	9 (6.3%)	
**Coronary heart disease**			>0.999
0	217 (99.5%)	144 (100.0%)	
1	1 (0.5%)	0 (0.0%)	
**Location**			0.644
1	18 (8.3%)	14 (9.7%)	
2	111 (50.9%)	78 (54.2%)	
3	89 (40.8%)	52 (36.1%)	
**cTNM**			0.962
2	80 (36.7%)	52 (36.1%)	
3	123 (56.4%)	81 (56.3%)	
4	15 (6.9%)	11 (7.6%)	

Mean ± SD; *n* (%).

### 3.2 LASSO regression variable screening and univariate and multivariate analysis

**FIGURE 1:**
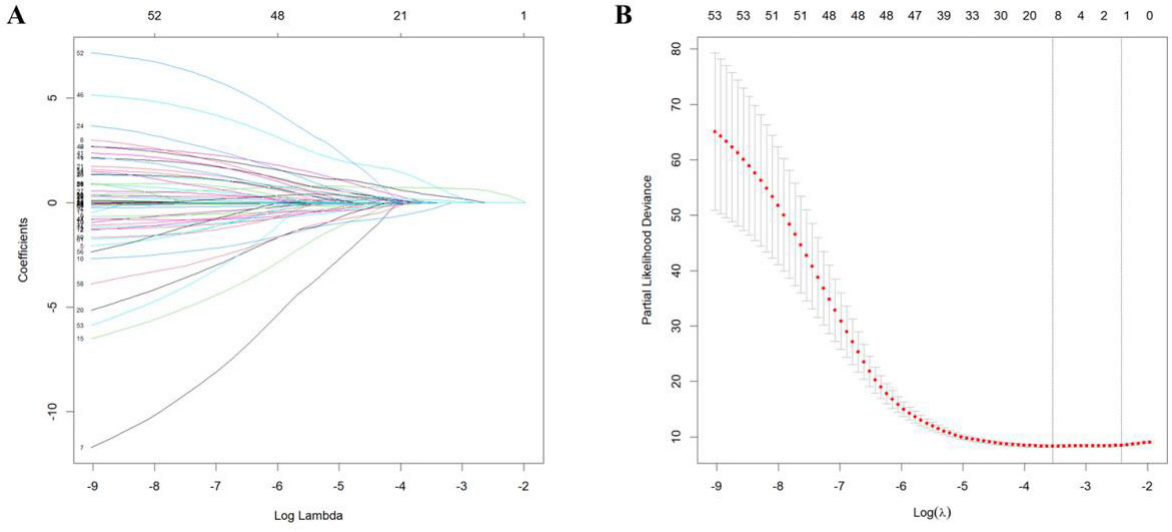
Least absolute shrinkage and selection operator regression curves. **(A)** Regression coefficient curve vs. log (λ). **(B)** Mean squared error curve vs. log (λ).

To address potential overfitting risks associated with high-dimensional covariates and multicollinearity issues, LASSO regression was implemented for feature selection among 41 candidate variables in the training cohort. This regularization procedure, optimized through 10-fold cross-validation, retained 10 variables at the λ threshold corresponding to minimal MSE and two variables under the λ + 1 SE criterion (Figure 1). To include more variables and improve model performance, we selected the minimum mean square error (λ value = 0.02898) for variable screening. LASSO regression identified the 10 most significant variables: smoking history, drinking history, diarrhea, number of lymph nodes dissected, number of lymph node dissection stations, pathological N (pN), pathological TNM (pTNM) TRG, nerve invasion, and arrhythmia (Figure 1).

### 3.3 Univariate and multivariate analyses of screening variables

**FIGURE 2:**
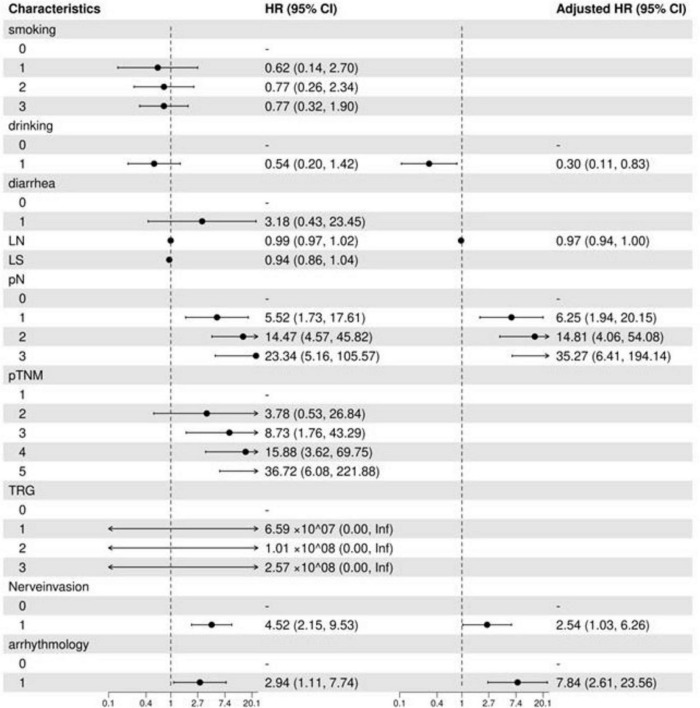
Filtered variable forest plot. LN, number of lymph nodes dissected; LS, number of lymph node dissection stations; TRG, tumor regression grade; OR, odds ratio; CI, confidence interval.

Univariate analysis revealed that pN staging [pN1: hazard ratio (HR) = 5.52 (1.73–17.61), pN3: HR = 23.34 (5.16–105.57)], and neuroinvasion [HR = 4.52 (2.15–9.53)] significantly increased the risk of death (all *p* < 0.05). Multivariate regression analysis using backward stepwise selection identified pN [pN1: HR = 6.25 (1.94–20.15), pN3: HR = 35.27 (6.41–194.14)], neurological invasion [HR = 2.54 (1.03–6.26), *p* = 0.043], and postoperative arrhythmia [HR = 7.84 (2.61–23.56), *p* = 0.002] as independent risk factors for increased mortality (Table 2). A forest plot was generated to visualize effect sizes of the filtered variable (Figure 2).

**TABLE 2:** Univariate and multivariate analysis of influencing factors.

Characteristic	Univariable					Multivariable				
	*N*	Event *N*	HR	95% CI	*p*-Value	*N*	Event *N*	HR	95% CI	*p*-Value
**Smoking**					0.874					
0	99	15	–	–						
1	19	2	0.62	0.14, 2.70						
2	39	4	0.77	0.26, 2.34						
3	61	7	0.77	0.32, 1.90						
**Drinking**					0.182					0.011
0	155	23	–	–		155	23	–	–	
1	63	5	0.54	0.20, 1.42		63	5	0.30	0.11, 0.83	
**Diarrhea**					0.336					
0	216	27	–	–						
1	2	1	3.18	0.43, 23.45						
**LN**	218	28	0.99	0.97, 1.02	0.671	218	28	0.97	0.94, 1.00	0.018
**LS**	218	28	0.94	0.86, 1.04	0.247					
**pN**					<0.001					<0.001
0	121	4	–	–		121	4	–	–	
1	60	10	5.52	1.73, 17.61		60	10	6.25	1.94, 20.15	
2	32	11	14.47	4.57, 45.82		32	11	14.81	4.06, 54.08	
3	5	3	23.34	5.16, 105.57		5	3	35.27	6.41, 194.14	
**pTNM**					<0.001					
1	94	2	–	–						
2	27	2	3.78	0.53, 26.84						
3	35	6	8.73	1.76, 43.29						
4	57	15	15.88	3.62, 69.75						
5	5	3	36.72	6.08, 221.88						
**TRG**					<0.001					
0	52	0	–	–						
1	51	4	6.59 × 10^7^	0.00, Inf						
2	44	5	1.01 × 10^8^	0.00, Inf						
3	71	19	2.57 × 10^8^	0.00, Inf						
**Nerve invasion**					<0.001					0.043
0	176	15	–	–		176	15	–	–	
1	42	13	4.52	2.15, 9.53		42	13	2.54	1.03, 6.26	
**Arrhythmology**					0.053					0.002
0	203	23	–	–		203	23	–	–	
1	15	5	2.94	1.11, 7.74		15	5	7.84	2.61, 23.56	

HR, hazard ratio; CI, confidence interval.

### 3.4 Nomogram creation and prediction model development

**FIGURE 3:**
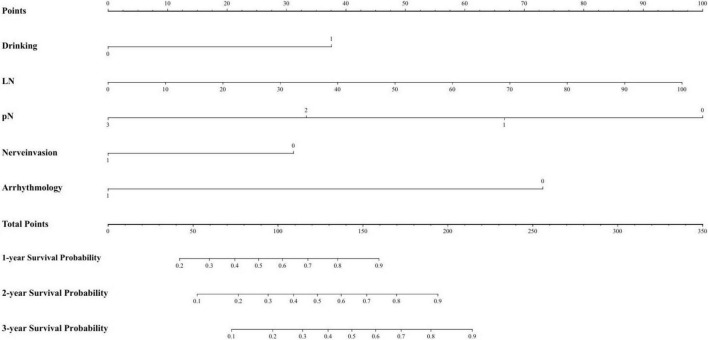
Nomogram of filtered variables.

Based on the regression coefficients from the multivariate analysis, a risk stratification model was developed to identify high-risk groups. The specific parameters of the model are provided in the checklist. The risk score was calculated using this model, and the 75th percentile of the risk score (cutoff = 0.962) was used to categorize the training set into high-risk and low-risk groups. Figure 3 presents the nomogram, and Table 3 provides the checklist of model parameters.

**TABLE 3:** Checklist of model parameters.

Variable	Parameter	Condition
Drink	−1.212	Positive
LN	−0.031	Continuous variables
pN	1.833	pN = 1
	2.695	pN = 2
	3.563	pN = 3
Nerve invasion	0.933	Positive
Arrhythmology	2.060	Positive

### 3.5 Predictive modeling and validation

**FIGURE 4:**
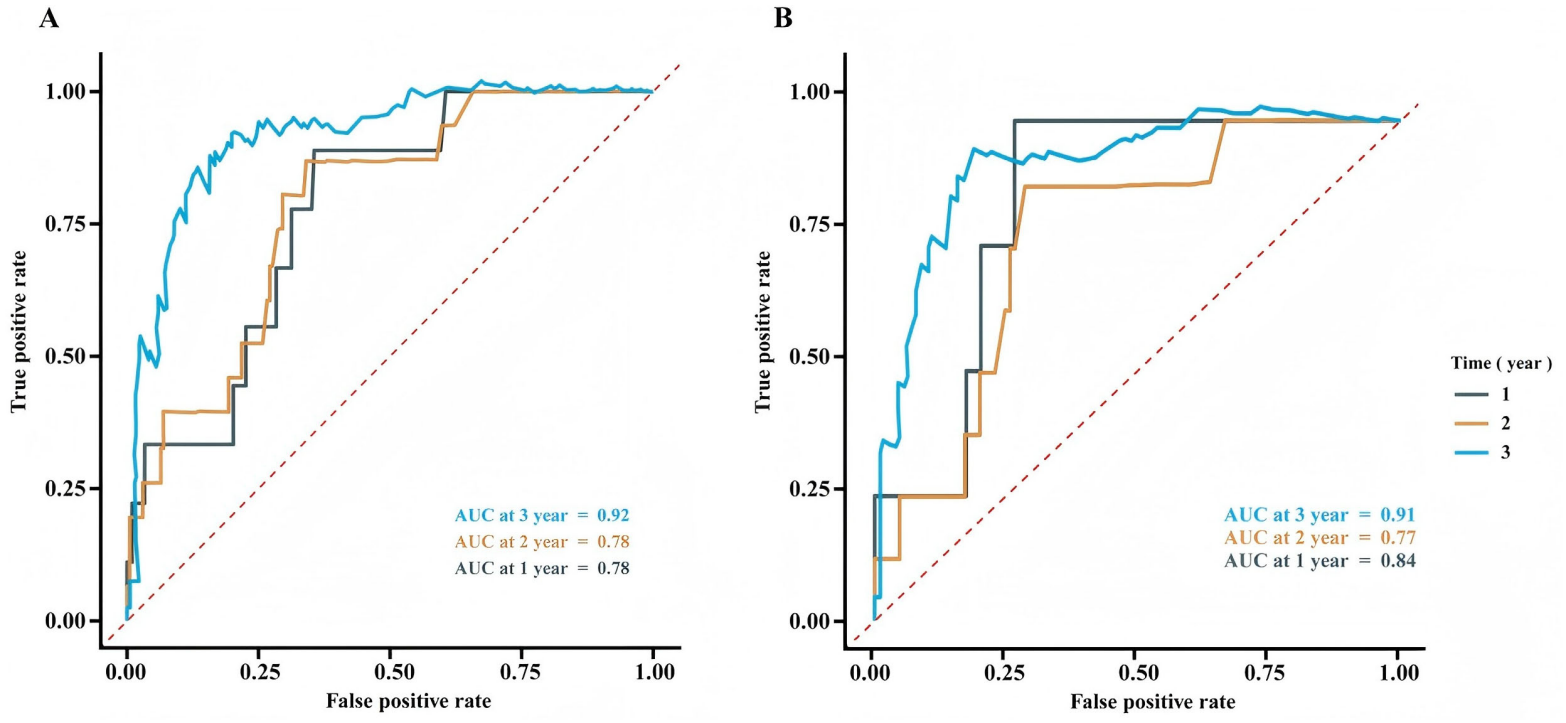
Receiver operating characteristic curve analysis for the training set **(A)** and validation set **(B)**.

The predictive performance of the prognostic model at various time points (1, 2, and 3 years) was assessed using ROC curve analysis for both the training set (Figure 4A) and the validation set (Figure 4B). Figure 4 shows the ROC curves for both sets.

### 3.6 Comparison of overall survival and relapse-free survival between low-risk and high-risk groups of patients

**FIGURE 5:**
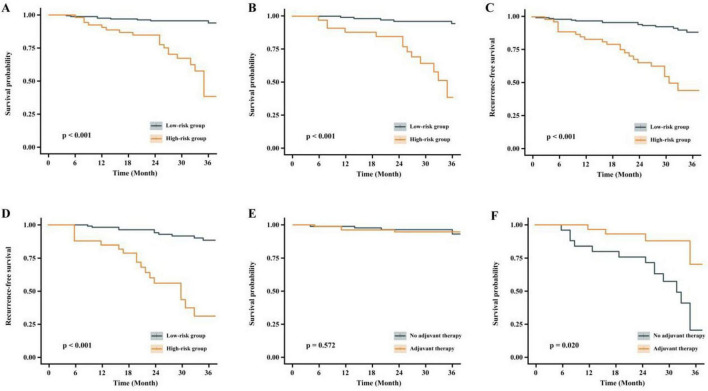
Comparison of OS and RFS between low-risk and high-risk groups. **(A)** Overall survival comparison between low-risk and high-risk groups (training set). **(B)** Overall survival comparison between risk groups (validation set). **(C)** Disease-free survival analysis in training set. **(D)** Disease-free survival analysis in validation set. **(E)** Postoperative adjuvant chemotherapy administration in low-risk group. **(F)** Postoperative adjuvant chemotherapy administration in high-risk group.

The validation set was also divided into high-risk and low-risk groups based on the same cutoff point. Patients treated with minimally invasive esophagectomy (MIE) after nICT were categorized into low-risk and high-risk groups using predictive modeling. Figure 5A shows the comparison of overall survival (OS) between the low-risk and high-risk groups in the training set, while Figure 5B represents the comparison of OS between the two groups in the validation set. Figures 5C, D depict the disease-free survival for the training and validation sets, respectively. Figure 5E indicates whether the low-risk group received postoperative adjuvant chemotherapy, and Figure 5F shows whether the high-risk group received postoperative adjuvant chemotherapy. The survival analysis revealed a significant difference in 3-year OS and relapse-free survival (RFS) between the low-risk and high-risk groups. However, there was no significant difference in the OS or RFS when comparing the low-risk group with or without postoperative adjuvant chemotherapy. In the high-risk group, patients who received postoperative adjuvant therapy had a survival benefit compared to those who did not receive it. Figure 5 represents the comparison of OS and RFS in the low-risk and high-risk groups.

## 4 Discussion

### 4.1 Primary prognostic factors

In this study, we systematically evaluated the survival risk factors in patients with locally advanced ESCC treated with nICT followed by surgery. A prognostic model was constructed, and univariate and multivariate Cox regression analyses identified pN stage, nerve invasion and postoperative arrhythmia as independent risk factors for survival.

Our findings demonstrated a strong correlation between pN staging and patient prognosis (adjusted HR for pN3 = 35.27). Lymph node metastasis is a primary route of esophageal cancer dissemination, and postoperative pathological N staging serves as an indicator of lymph node involvement. Several studies have reported a significant association between pN stage and prognosis (12, 13), with poorer survival outcomes in patients with positive lymph node metastasis (14, 15).

Nerve invasion, as indicated by postoperative pathology, refers to cancer cell infiltration into the perineural space and is considered a key marker of tumor aggressiveness (16). In our study, univariate analysis showed that nerve invasion significantly increased the risk of death (HR = 4.52, *p* < 0.05), though this effect was attenuated in multivariate analysis (HR = 2.54), suggesting that its impact may be partially mediated by other factors, such as lymph node metastasis. Previous studies have also shown that nerve invasion is strongly associated with survival outcomes in esophageal cancer and is linked to poorer prognosis (16, 17). Furthermore, nerve invasion may facilitate tumor cell dissemination to lymph nodes via perineural space, contributing to disease progression (18).

Postoperative arrhythmia is a common complication following esophageal cancer surgery. Kashiwagi et al. (19) reported an 18% incidence of postoperative atrial fibrillation in these patients. In our study, postoperative arrhythmia was identified as an independent risk factor for poor prognosis. However, limited research exists on the long-term impact of postoperative arrhythmia on survival. While it may not directly influence long-term outcomes, postoperative arrhythmia can prolong hospitalization and negatively affect short-term recovery (19, 20).

### 4.2 Unexpected findings and their implications

In this study, alcohol consumption was considered a potential protective factor (HR = 0.30, 95% CI: 0.11–0.83). While alcohol consumption is typically recognized as a cancer risk factor in previous studies (21) and is classified as a class I carcinogen by the World Health Organization (22), this study’s results contradict the existing literature. The authors attribute this discrepancy to the retrospective nature of the study, which may introduce sample selection bias. Therefore, these findings should be interpreted with caution and validated further through prospective cohort studies or relevant basic research to avoid misleading clinical decision-making. The alcohol variable in this model only reflects the observational association within a specific cohort, and cannot be used to justify abstaining from alcohol. Clinical decisions must strictly adhere to the WHO’s cancer prevention recommendations.

Tumor regression grade has been associated with postoperative tumor recurrence, with better tumor regression generally correlating with improved prognosis for patients who have undergone neoadjuvant therapy (23). Although the HR value for TRG classification was high in this study (TRG1: HR = 6.59), the confidence interval included “Inf,” indicating that the model’s estimation may be limited by extreme values or small sample size. TRG is typically used to assess the tumor’s response to neoadjuvant therapy, but its instability in this study aligns with other research suggesting that patients who achieve a PCR after neoadjuvant therapy may still face postoperative recurrence (24). The authors believe individual differences in response to neoadjuvant therapy play a role, and while TRG reflects the tumor’s response to treatment, it cannot fully account for individual variability. Expanding the sample size in future studies will be necessary to clarify its clinical value.

In addition to TRG, postoperative pathologic staging is another important clinical indicator following neoadjuvant therapy. The prognosis of esophageal cancer is closely linked to postoperative pathological staging. pTNM staging is considered the gold standard for assessing prognosis after surgery (25). While pTNM staging was included in this study, it showed an increasing risk in univariate analysis (pTNM5: HR = 36.72) but was not incorporated into the multivariate model. This may be due to multicollinearity with pN staging. Future studies should employ stratified analysis methods to better understand the independent contributions of different staging systems.

### 4.3 Performance and clinical application of the prognostic model

The development of a postoperative recurrence risk model is a crucial aspect of precise, full-course tumor treatment. An effective recurrence risk model can aid in formulating individualized treatment strategies, optimizing follow-up protocols, assessing prognosis, and facilitating clinical communication. Additionally, it plays a significant role in the design of clinical research (26–29). In this study, the results showed that 3 years after surgery, the AUCs of the training set and validation set were 0.92 and 0.91, respectively. The close similarity between these values suggests the model has strong generalizability in predicting 3-year survival. This may be attributed to the fact that long-term prognosis is largely dependent on stable pathological features, such as pN staging and neurological invasion, which were successfully captured as core risk factors by the model. At 1 and 2 years post-surgery, the AUCs for the training set were both 0.78. In the validation set, the AUC for 1 year was slightly higher at 0.84 but decreased to 0.77 at 2 years. The higher 1-year AUC in the validation set compared to that of the training set suggests that the model did not overfit the 1-year prediction and may be more sensitive to early risk stratification in the validation cohort. However, the slight decrease in the 2-year AUC (from 0.78 to 0.77) warrants attention, as it may be related to the model’s limited ability to incorporate dynamic factors such as postoperative complications and treatment response.

In this study, patients who underwent MIE after nICT were categorized into low-risk and high-risk groups using predictive modeling. The 3-year OS and RFS were plotted separately for the training and validation sets. The results revealed significant differences in OS and RFS between the two groups in both sets. Regarding the selection of postoperative adjuvant therapy strategy, the low-risk group showed no significant benefit from postoperative adjuvant therapy. In contrast, the high-risk group demonstrated superior OS when postoperative adjuvant therapy was administered. This suggests that patients in the low-risk group could be exempt from postoperative adjuvant therapy, whereas those in the high-risk group may benefit from an aggressive postoperative adjuvant therapy regimen. Similar findings were reported by Francis et al. (13), who analyzed 2,045 patients with persistent lymph node positivity (pN+) after undergoing nCRT and esophagectomy. Their study indicated that postoperative aCT treatment was associated with an HR for death of 0.78 (95% CI: 0.62–0.97; *p* = 0.032), suggesting a significant survival benefit with aCT (13). The choice of postoperative adjuvant therapy for patients with esophageal cancer and pathological evidence of nerve invasion is critical. Several studies have evaluated the impact of neoadjuvant chemotherapy and postoperative adjuvant chemotherapy on long-term survival (30, 31). However, the optimal treatment regimen for patients with positive nerve invasion remains uncertain, necessitating further research to clarify the most effective approach.

Finally, it should be noted that the current model lacks formal calibration assessment. Although risk stratification and clinical utility analyses support its robustness, future validation with calibration plots and goodness-of-fit tests is warranted.

## 5 Conclusion and future directions

In summary, for ESCC treated with nICT followed by MIE, patients with a high pN stage or pathological evidence of nerve invasion require closer postoperative imaging surveillance and a shortened follow-up interval to enable early detection of metastatic lesions. Additionally, intensive adjuvant therapy should be considered to improve long-term survival outcomes.

## Data Availability

The original contributions presented in this study are included in this article/supplementary material, further inquiries can be directed to the corresponding author.
